# Primary Eosinophilic Colitis Manifesting As Acute Appendicitis: A Case Report and Literature Review

**DOI:** 10.7759/cureus.98176

**Published:** 2025-11-30

**Authors:** Eimante Raupelyte, Mark Youssef, Christopher Delaney, Michael Green, Ivan Tomasi

**Affiliations:** 1 Department of General Surgery, Guy’s and St Thomas’ Hospitals NHS Trust, London, GBR; 2 Department of Pathology, Guy’s and St Thomas’ Hospitals NHS Trust, London, GBR

**Keywords:** acute eosinophilic appendicitis, appendicectomy, gastrointestinal surgery, primary eosinophilic colitis, uncomplicated appendicitis

## Abstract

Eosinophilic colitis (EC) is a rare inflammatory disorder characterised by eosinophilic infiltration of the gastrointestinal tract, often resulting in varied clinical presentations. The role of the appendix in such inflammatory processes remains underexplored. We describe the case of a 48-year-old male who presented with acute abdominal pain, initially managed as uncomplicated appendicitis. He had a history of chronic diarrhoea and no atopic background. Histological examination of the resected appendix revealed eosinophilic infiltration without evidence of acute inflammation. Subsequent investigations ruled out secondary causes, leading to a diagnosis of primary EC. This case highlights the potential for eosinophilic appendicitis to mimic acute appendicitis clinically and the need for postoperative evaluation to exclude systemic eosinophilic gastrointestinal disorders. This represents, to our knowledge, a case of primary EC resolving after appendicectomy. We hypothesise that the appendix played a contributory role in the maintenance of chronic mucosal inflammation in this patient.

## Introduction

Eosinophilic gastroenteritis was first described by Kaijser in 1937, yet its true prevalence remains uncertain due to the absence of standardised diagnostic criteria [1]. To date, approximately 300 cases have been reported in the literature. The condition typically affects individuals between 30 and 40 years of age and is often associated with a history of atopic disease or food allergies. Histologically, eosinophilic gastrointestinal disease can present either as localised or diffuse eosinophilic infiltration within the gastrointestinal tract [2].

Clinically, eosinophilic gastrointestinal disorders exhibit two major patterns of presentation. Patients may experience an acute onset of symptoms, such as abdominal pain, vomiting, and gastrointestinal bleeding, or a more chronic course characterised by diarrhoea, weight loss, and malabsorption [2]. The specific clinical manifestations largely depend on the depth and location of eosinophilic infiltration within the bowel wall. Mucosal involvement tends to result in symptoms such as vomiting, diarrhoea, rectal bleeding, and malabsorption, while muscular layer involvement often leads to bowel obstruction-like symptoms. Serosal involvement, though less common, can manifest with ascites formation [2].

Eosinophilic colitis (EC) is an uncommon manifestation of eosinophilic gastrointestinal disorders [2]. It can typically be seen in healthy infants presenting with bloody diarrhoea or chronic relapsing colitis in young adults.

Acute eosinophilic appendicitis (AEA) is an uncommon clinical entity, with few cases reported in the medical literature. In most instances, AEA presents similarly to uncomplicated acute appendicitis, making preoperative differentiation challenging. Furthermore, while appendicectomy often addresses the acute symptoms, identification of eosinophilic infiltration on histopathological examination necessitates a broader evaluation to exclude systemic eosinophilic gastrointestinal disorders or secondary causes such as parasitic infection, allergy, vasculitis, or mastocytosis. The diagnosis of eosinophilic gastrointestinal diseases is often challenging and requires a high index of suspicion, supported by the exclusion of secondary causes through clinical, radiological, and histopathological evaluation [3].

We present a rare case of primary EC manifesting with symptoms initially suggestive of acute appendicitis, which resolved following laparoscopic appendicectomy. This case report is prepared following the SCARE criteria.

## Case presentation

A 48-year-old male of Brazilian origin presented to the emergency department (ED) with a three-day history of generalised abdominal pain radiating to both flanks and the testes. Symptoms were associated with a right-sided limp and three episodes of non-bilious vomiting. He reported recent ingestion of Brazilian meats, salad, rice, and beans, though no other individuals in his company developed symptoms. Dysuria was noted, but urinalysis was unremarkable.

The patient had a ten-year history of chronic diarrhoea, characterised by up to eight bowel movements per day of type 7 stool as per the Bristol Stool Chart [4], without blood or mucus. His past medical history included two prior hospitalisations for diarrhoea and rectal bleeding, a history of nephrolithiasis, and a prior cardiac arrest.

Occupational history included construction work, baking, and fishing. He denied smoking and reported moderate alcohol intake. Travel history included stays in Brazil, the USA, and Portugal. Notable allergies included latex sensitivity (contact dermatitis).

On presentation, he was hemodynamically stable and afebrile, with a NEWS2 (National Early Warning Score 2) score of 0 [5]. Physical examination revealed right iliac fossa tenderness without peritonism and mild bilateral flank tenderness. Scrotal examination was normal. Baseline laboratory investigations are summarised in Table 1. An important finding of note was the mild eosinophilia observed.

**Table 1:** Patient Investigation Results on First Presentation

Laboratory Investigations	Patient Results	Reference Range
White blood cells	8.5 ×10⁹/L	4 - 11 × 10⁹/L
Neutrophils	4.2 ×10⁹/L	2 - 7.5 × 10⁹/L
Eosinophils	0.6 ×10⁹/L	0.04 - 0.44 × 10⁹/L
C-reactive protein	5 mg/L	<5 mg/L
Lactate	1.45 mmol/L	0.6 - 1.8 mmol/L
SARS-CoV-2 PCR	Negative	N/A

**Figure 1:**
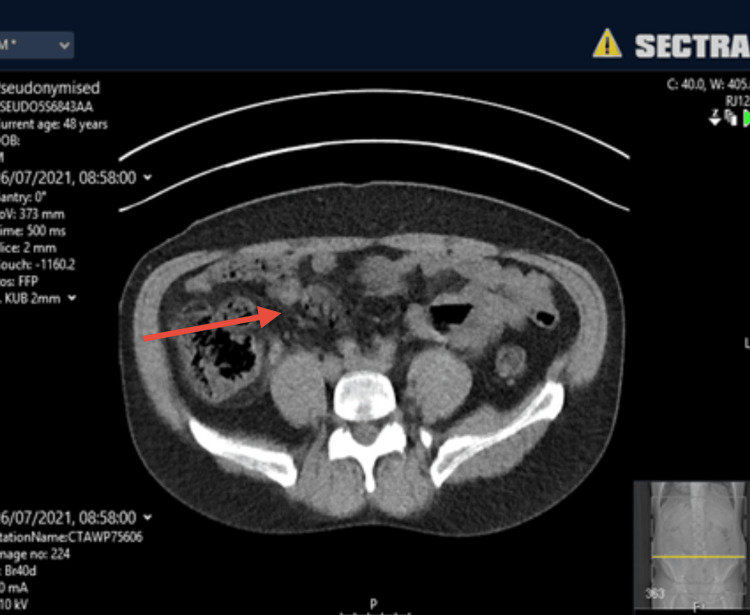
CT KUB image illustrating appendicitis (Axial view) The red arrow indicates where appendicitis can be seen on the image. CT KUB: computed tomography (CT) scan of the kidneys, ureters, and bladder

**Figure 2:**
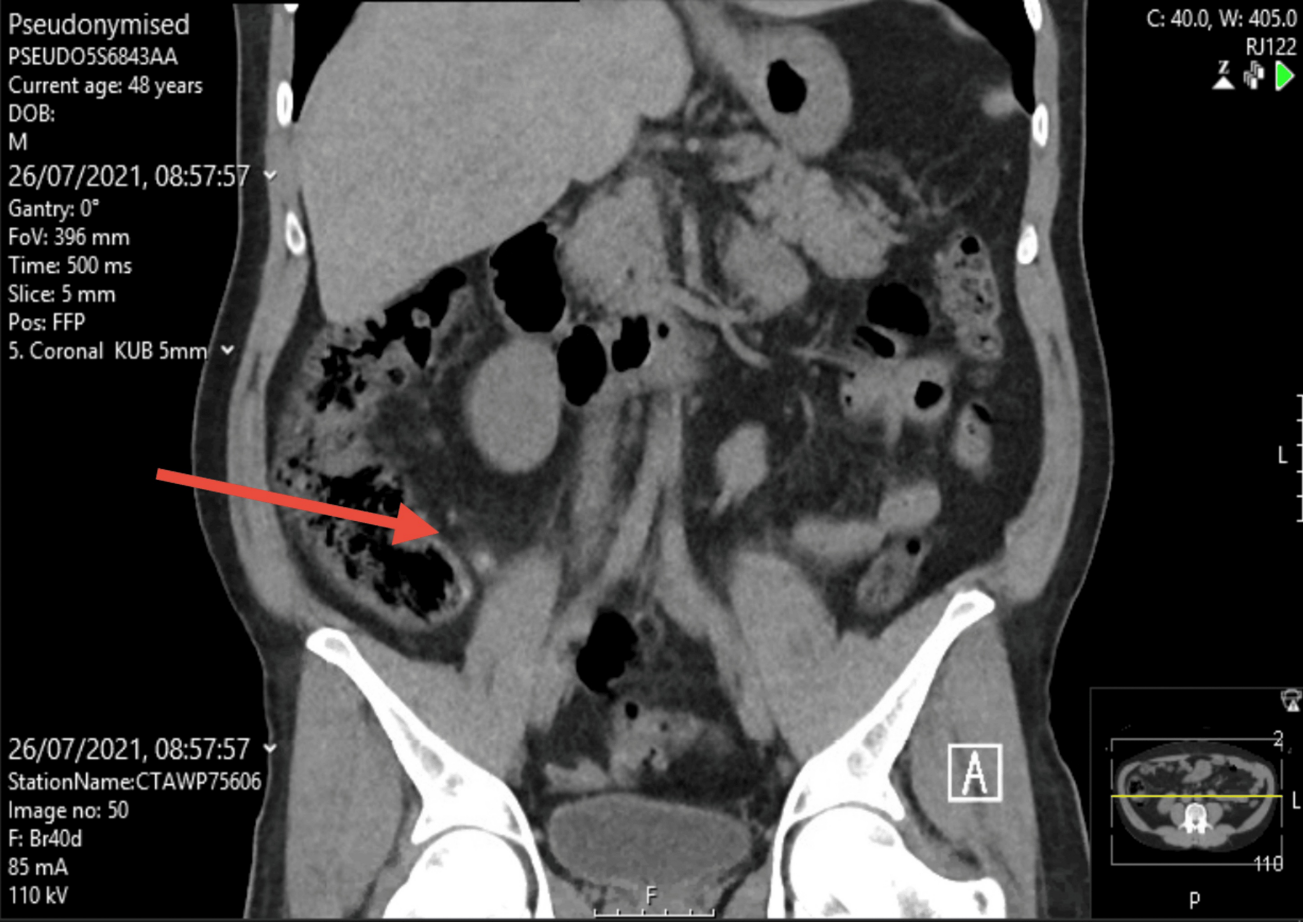
CT KUB image illustrating appendicitis (Coronal view) The red arrow indicates where appendicitis can be seen on the image. CT KUB: computed tomography (CT) scan of the kidneys, ureters, and bladder

Computed tomography of the kidneys, ureters, and bladder (CT KUB) imaging demonstrated uncomplicated appendicitis (Figures 1-2). Given the clinical findings, he was managed conservatively with oral antibiotics and discharged with safety-net advice and outpatient follow-up. However, symptoms of radiating pain to the testes and a limp are unusual clinical features of appendicitis. 

The patient re-presented three days later with worsening right iliac fossa pain. Blood tests were repeated at re-presentation and summarised in Table 2.

**Table 2:** Patient Investigation Results on Re-presentation

Laboratory Investigations	Patient Results	Reference Range
White blood cells	7.9 ×10⁹/L	4 - 11 × 10⁹/L
Neutrophils	3.6 ×10⁹/L	2 - 7.5 × 10⁹/L
Eosinophils	0.4 ×10⁹/L	0.04 - 0.44 × 10⁹/L
C-reactive protein	3 mg/L	<5 mg/L
Lactate	<1.8 mmol/L	0.6 - 1.8 mmol/L

Due to clinical deterioration, a laparoscopic appendicectomy was performed. Intra-operatively, the appendix appeared retrocecal, enlarged, and contained a faecolith. No abnormalities were seen in the small or large bowel.

Postoperatively, the patient recovered well and was discharged on postoperative day 1.

**Figure 3:**
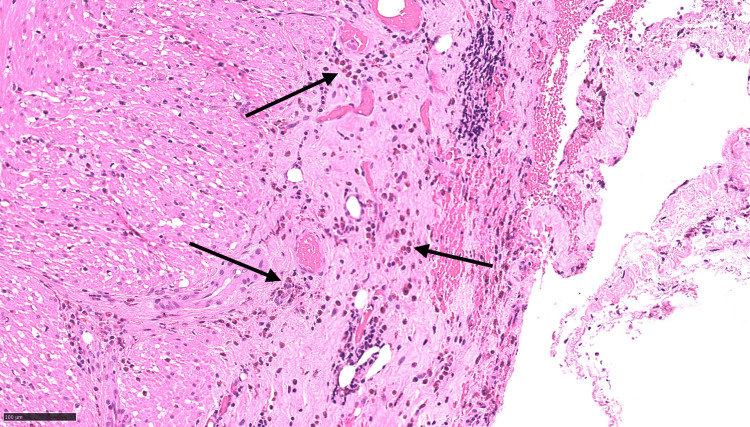
Black arrows indicate eosinophilic infiltration in the subserosa of the appendix on histopathological staining Eosinophilic infiltration is demonstrated by the red granules in the cytoplasm of cells, using the black arrows. Images visualised using haematoxylin and eosin (H&E) staining and x100 power microscopy.

**Figure 4:**
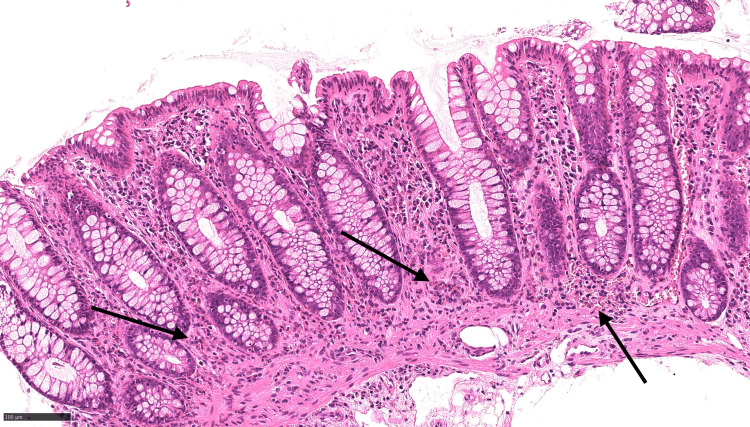
Black arrows indicate eosinophilic infiltration of the colon mucosa on histopathological staining Eosinophilic infiltration demonstrated by the red granules in the cytoplasm of cells using the black arrows. Images visualised using H&E staining and ×100 power microscopy.

**Figure 5:**
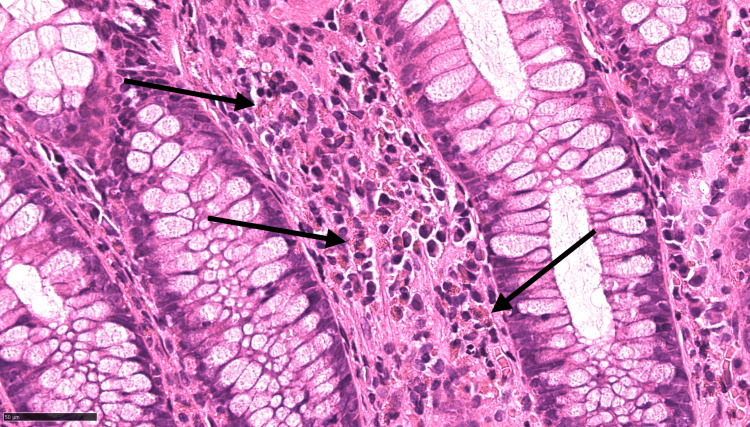
Black arrows indicate eosinophilic infiltration of the colon mucosa on histopathological staining Eosinophilic infiltration demonstrated by the red granules in the cytoplasm of cells using the black arrows. Images visualised using H&E staining and ×400 power microscopy.

Histopathological examination revealed a 50 mm appendix with submucosal fibrosis and patchy eosinophilic infiltration of all layers, without evidence of acute inflammation, granulomas, vasculitis, parasites, dysplasia, or malignancy (Figures 3-5). 

At one-month follow-up, the patient reported significant improvement in diarrhoea, with bowel movements normalising to once daily (Bristol Stool Chart type 4) by three months postoperatively [4]. Additionally, he was able to consume dairy products without gastrointestinal symptoms, suggesting no history of lactose intolerance. He was also reviewed in the clinic six months postoperatively and confirmed ongoing resolution of gastrointestinal symptoms.

Further investigations were arranged to assess for potential causes of the patient’s gastrointestinal symptoms. The results are summarised in Table 3. Causes such as coeliac disease, thyroid abnormalities and parasitic infection were ruled out. An abdominal ultrasound was performed to assess for mast cell disease. No hepatic or splenic pathologies were detected. A colonoscopy was arranged, which also showed no significant abnormalities. Given the exclusion of secondary causes, a diagnosis of primary EC was established.

**Table 3:** Further Patient Investigations and Results to Identify Causes of Gastrointestinal Symptoms

Investigations	Reference Range	Patient Results
Stool microscopy	N/A	Negative
Faecal calprotectin	<100 µg/g	46 µg/g
Thyroid-stimulating hormone (TSH)	0.3–4.5 pmol/L	0.78 pmol/L
Tissue transglutaminase antibodies	<7 U/ml	1 U/mL
Tryptase	0-12.9 µg/L	6 µg/L

## Discussion

Primary EC is a rare condition characterised by tissue eosinophilia [3]. An American study estimated the prevalence of EC at 3.3/100,000, and another study of 1621 appendectomies found 10 to have eosinophilic infiltrate, a prevalence of 0.62% [6,7].

The aetiology is unclear, but one proposed theory is food hypersensitivity (FH) [8]. In contrast, secondary EC has many known causes, including medications (e.g. Clozapine), helminthic infections, inflammatory bowel disease, and autoimmune disease (e.g. scleroderma, Churg-Strauss syndrome), amongst other causes [2].

There are two known mechanisms of FH : IgE and non-IgE-mediated responses. In adulthood, T cell-mediated response is proposed to be the most common mechanism, as IgE-mediated allergic responses are more commonly present in childhood [2]. Although the mechanisms have been proposed, the aetiology remains unclear. There is a strong association between atopy and EC; however, removal of known allergens from the diet is not often successful in improving symptoms of EC [2,9].

Studies have linked the appendix to the development of inflammatory bowel diseases such as ulcerative colitis (UC) [10]. The appendix has specific populations of T and B cells, which are thought to play a role in initiating the colorectal inflammation of UC [10]. This suggests that the appendix may have played a role in the development of the chronic diarrhoea reported in the above case. The improvement of symptoms following the removal of the appendix also points to it as the primary causative organ in this case. 

Other case reports have similar findings, with symptoms improving following removal of the eosinophilic appendix [11]. However, one study of histopathological findings following appendicectomy found a 16.7% recurrence of intestinal eosinophilia following appendicectomy [12]. 

A history of atopy is common in cases of EC, but one study of eight case reports of AEA concluded there was no history of atopy [13]. Another study of 120 appendicectomies suggested mast cell degranulation in the specimens was evidence that AEA is IgE mediated [14]. Many of the cases of AEA reviewed were secondary to parasite infestation, such as Taenia saginata, Strongyloides, and Schistosoma [15]. 

There are no specific diagnostic criteria for EC, although in cases of mucosal EC, endoscopy can reveal mucosal inflammation [3,16]. Eosinophilic infiltration of tissue is required for a diagnosis of EC to be confirmed on histopathology [17]. Our patient was found to have acute eosinophilic appendicitis, which was mimicking acute appendicitis. AEA, like EC, has no defined diagnostic criteria.

In EC, eosinophil infiltration can be found in mucosal, muscular and serosal layers, in order of prevalence [3]. Mucosal disease presents with abdominal pain, diarrhoea, nausea, vomiting which can be difficult to distinguish from other GI diseases such as suppurative appendicitis, as in our case, who was initially treated unsuccessfully with a course of antibiotics [3]. Muscular involvement leads to bowel wall thickening and symptoms mimicking obstruction. AEA appears to present most commonly with muscular eosinophil infiltration [13].

Clinical manifestations of EC are varied. Many patients can remain asymptomatic while others can experience non-specific signs such as abdominal pain, nausea, weight loss and fever [18]. Symptoms can be correlated to the extent of disease in the various layers. If there is mucosal involvement, anaemia secondary to GI bleeding, diarrhoea or enteropathy can be observed. Muscular or serosal involvement can rarely present as bowel obstruction and ascites [18].

In primary EC, medical management is similar to that of inflammatory bowel diseases. Steroids are used to induce remission and steroid-sparing agents for maintenance therapy [3]. Much of the knowledge regarding the treatment of EC is derived from case reports, and there are no defined guidelines for treatment.

The use of steroids to treat AEA has not been documented in case reports. Primarily, the treatment is appendicectomy. When presented with a case of acute appendicitis not responding to the antibiotics, surgical intervention is likely to be the next step in treatment; hence, cases of AEA are diagnosed following appendicectomy [11,15]. 

Biological agents are being trialled in cases of eosinophilic oesophagitis (EO). Reslizumab appears to improve symptoms, although tissue eosinophilia count remains high [19]. Treatments targeting IgE and non-IgE mediated responses appear to generate symptomatic benefit for EO, illustrating perhaps both pathways play a role in the condition [20]. Due to the similarities between these conditions, biologic agents may present future treatments for EC.

There is a lack of randomised controlled trials for the diagnosis and treatment of EC/AEA. This is likely due to the rarity of the condition. It is also noted that the case reported is unique in that appendicectomy was associated with complete resolution of chronic diarrhoea. Without previous similar cases to compare this to, it is difficult to say for certain that the two variables are correlated.

## Conclusions

We report the first documented case of primary EC resolving following appendicectomy. This case illustrates the variable presentations of eosinophilic gastrointestinal disease and emphasises the need for thorough postoperative evaluation to exclude systemic disease.

Further studies are warranted to elucidate the immunological role of the appendix in chronic gastrointestinal inflammation. It is the hope that this report will add to the growing library of literature on this topic and improve understanding of its presentation and treatment.
